# Exosomes Released by Bone Marrow Mesenchymal Stem Cells Attenuate Lung Injury Induced by Intestinal Ischemia Reperfusion via the TLR4/NF-κB Pathway

**DOI:** 10.7150/ijms.35369

**Published:** 2019-08-14

**Authors:** Jianpei Liu, Tufeng Chen, Purun Lei, Xiao Tang, Pinjie Huang

**Affiliations:** 1 Department of Gastrointestinal Surgery, The Third Affiliated Hospital of Sun Yat-sen University, Guangzhou, China, 510630.; 2 Department of Anesthesiology, The Third Affiliated Hospital of Sun Yat-sen University, Guangzhou, China, 510630.

**Keywords:** Mesenchymal stem cell, exosome, ischemia reperfusion, intestine, lung injury, Toll-like receptor 4.

## Abstract

***Purpose:*** Acute lung injury (ALI) is a primary component of multiple organ dysfunction syndromes triggered by intestinal ischemia-reperfusion (IIR) which results in high mortality. Existing treatment options remain unsatisfactory. Mesenchymal stem cells (MSCs) have shown considerable promise as a biological therapy for ALI in preclinical studies. However, there are many limitations to stem cell treatment. This study aimed to investigate whether MSC-derived exosomes, a non-cellular alternative, are able to act in a protective capacity similar to that of MSCs for ALI triggered by IIR in a rat model and to explore the underlying mechanisms.

***Methods:*** The IIR model involved occlusion of the superior mesenteric artery of a rat for 75 min then reperfusion for 20 h. Rats then received an intravenous injection of either bone marrow-derived MSCs or MSC-derived exosomes. Pathologic alteration of lung tissue, levels of pro-inflammatory cytokines, apoptotic proteins and TLR4/NF-κB signaling were measured to evaluate the therapeutic effect of treatment with either MSCs or exosomes.

***Results:*** Manifestations of acute lung injury after IIR were observed as edema and hemorrhage of alveoli and mesenchyme, and inflammatory cell infiltration. MSCs and MSC-derived exosomes both attenuated IIR-induced lung damage by decreased apoptosis and inflammation accompanied by down-regulation of TLR4 and NF-κB expression.

***Conclusions:*** MSC-derived exosomes provide protection similar to that of MSCs against IIR-induced ALI via inhibition of TLR4/NF-κB signaling, suggesting that a potential strategy against IIR-mediated acute lung injury could be therapy with exosomes as a non-cellular alternative to MSC transplantation.

## Introduction

Intestinal ischemia-reperfusion (IIR) injury is a serious but common clinical occurrence caused by a number of pathophysiological factors, including superior mesenteric artery occlusion, abdominal and thoracic vascular surgery, cardiopulmonary bypass or small intestine transplantation, resulting in severe local and remote tissue injury and subsequent organ dysfunction 1, 2. Acute lung injury (ALI), which can manifest in clinic as acute respiratory distress syndrome (ARDS) is a primary component of multiple organ dysfunction syndromes (MODS) triggered by IIR which results in high mortality, of approximately 40% 3. Several studies have demonstrated that oxidative stress and inflammation play critical roles in damage to pulmonary cells, leading to loss of alveolocapillary membrane integrity and impaired surfactant function triggered by small intestinal ischemia reperfusion 4, 5. However, the precise mechanism remains to be elucidated, with only a limited number of pharmacological treatment options available to ameliorate morbidity in patients with ARDS 6. Thus, a major aim of this research is to develop effective therapies for lung injury induced by IIR through elucidation of the mechanisms of the condition.

Mesenchymal stem cell (MSC) research has expanded greatly since the 1970s, the cells demonstrating great promise as a biological therapy for a diverse range of diseases in preclinical studies 7. MSCs are stem cells that remain in adult tissues, having the capability to undergo unlimited amplification and multipotent differentiation 8. Interest in MSCs as a possible therapy stems largely from their ability to modulate the host immune response to injury and infection and promote repair following tissue injury 7, 9, 10. Although many clinical trials of MSCs have been predicated on the hypothesis that transplanted MSCs home and engraft into injured tissues prior to differentiating into cells that replace damaged tissue, it has become apparent that engraftment and differentiation at sites of injury are unlikely to account for the therapeutic effects of MSC transplantation 10-12. There is increasing evidence that the therapeutic efficacy of MSCs is mediated by exosomes, small membrane vesicles 40-90 nm in diameter, originating from many cell types and acting as mediators of cell-to-cell communication 11, 13. Exosomes harbor a discrete set of proteins and RNA from their originating cell, implying that they have the potential for unique bioactivity and function 14. Compared with MSCs, cell-free exosomes are likely to be benign, not elicit intrinsic adverse effects or immune rejection 14. Recent studies indicate that MSC-derived exosomes are efficacious in animal models of various tissue injuries 15. Despite the clear recognition that exosomes may elicit a novel paracrine mechanism in MSC-mediated tissue regeneration, there are no published reports about the therapeutic potential of exosomes secreted by MSCs for acute lung injury triggered by IIR.

The purpose of present study was to investigate whether MSC-derived exosomes exhibit a protective capacity similar to that of MSCs in lung injury triggered by intestinal ischemia-reperfusion in a rat model and to explore the underlying mechanisms.

## Materials and Methods

### Experimental model

This study was approved by the Institutional Animal Care and Use Committee of Sun Yat-Sen University in Guangzhou, China and complied with national guidelines for the treatment of animals. Forty healthy male adult Sprague-Dawley rats weighing 180-270g were raised on a basic diet for a week at a stable room temperature (25-27℃), illuminated from 8:00am to 8:00pm. Prior to surgery, all rats were fasted for 16 h with free access to water. The rats were randomly assigned to one of four groups and anesthetized using diethyl ether. In the IIR, MSC and MSC-EX groups, the abdomen was opened, the superior mesenteric artery (SMA) was identified and clamped for 75 min and then the incision was closed following removal of the clamp to initiate reperfusion. In the sham-operated group (SHAM group), the abdomen was opened and the SMA was isolated without clamping. Immediately following closure of the incision, all rats were resuscitated using 1.5 mL of normal saline injected subcutaneously. A total of 3 × 10^6^ MSCs suspended in 500 μL PBS (MSC group) or 5 - 10 μg of exosome protein (MSC-EX group) suspended in 500 μL PBS were injected into the tail vein of each rat. As controls, the same volume of PBS without exosomes was infused into the SHAM and IIR groups using the same route. After the specified 20h period of reperfusion, the ten rats in each group were sacrificed using large doses of intraperitoneal pentobarbital (200 mg/kg). Following confirmation of loss of righting reflex, cessation of heartbeat and breath were confirmed, thoracotomy was performed rapidly to collect lung samples.

### Isolation of rat MSCs

Bone marrow from the femoral and tibial cavities of Sprague-Dawley rats was flushed with DMEM (Gibco, Rockville, MD) containing 10% fetal bovine serum (FBS; Gibco) plus penicillin and streptomycin (100 U/mL and 0.1 mg/mL, respectively, Gibco), the suspension of cells then centrifuged (200 g, 5 min). The cells were then plated in flasks (200,000 cells/cm^2^). Non-adherent cells were removed after 48 h, the MSCs purified by virtue of their capacity to adhere strongly to plastic culture flasks. MSCs were used at passages 3-5 of for all experiments.

### Exosome isolation and purification

MSCs were cultured in media supplemented with 10% exosome-depleted FBS (FBS, Gibco). The depletion of bovine exosomes from FBS was achieved by ultracentrifugation at 100,000g for 70 min. Rat exosomes were collected from 24-hour culture in conditioned media through standard differential centrifugation steps. The cell culture supernatant was collected and the exosomes isolated by centrifugation at 2000 x g for 20 min then by pelleting using ultracentrifugation at 100,000 x g for 1 h at 4℃. Finally, the exosome pellet was washed in a large volume of PBS then resuspended in PBS. The exosomes were further purified by resuspending in 2.5 M sucrose in 25 mM HEPES buffer (pH 7.4). They were then subsequently loaded into the bottom of a SW41 tube. HEPES buffer (25mM) containing 2 M sucrose was carefully loaded on top of the exosomes followed by HEPES buffer (25 mM) containing 0.25 M sucrose to produce a discontinuous 2-0.25 M sucrose gradient. After spinning overnight at 100,000 x g in an SW41 swing rotor, 1 mL of each fraction was collected then centrifuged at 100,000 x g for 1 h. After aspirating the supernatant, the pellet was resuspended in PBS, the protein content quantified using a bicinchoninic acid (BCA) assay (Thermo Fisher Scientific Inc.) and then stored at -80℃ until required for use.

### Characterization of MSCs and exosomes

MSC phenotype was confirmed by detecting the presence of cells with a typical spindle-shaped appearance using electron microscopy and typical biomarkers detected using Western blot analysis, as described in the following section. Anti-CD90, anti-CD81, anti-CD63 and anti-TSG101 polyclonal antibodies (1:1000 dilution; Santa Cruz) were used for MSC characterization. The morphology and ultrastructure of the exosomes were ascertained using transmission electron microscopy.

### Lung histology

Tissue from the left lung was sectioned (4 μm) and stained with hematoxylin - eosin. The degree of lung injury was assessed using a scoring system as described by Derks *et al.*, that evaluated edema in the alveolar mesenchyme, edema in alveoli, intra-alveolar cell infiltration, alveolar hemorrhage and atelectasis 16. Each parameter was scored on a scale of 0-3. Pathological scores were assessed by an investigator who was blinded to initial research grouping.

### Wet to dry lung ratio

Five rats from each group were used to determine the wet-to-dry lung ratio (W/DR) as an indicator of pulmonary edema. The left lung was excised and immediately weighed using a precision balance and then re-weighed after being dried at 80℃ for 24 h in an oven. The left lungs of the remaining four animals of each group were used for histologic assessment. The right lungs were washed with cold saline and dried with filter paper then stored at -80℃ for further analysis.

### Enzyme-linked immunosorbent assay (ELISA)

Right lung tissue was homogenized in cold normal saline, and then centrifuged at 4000 r/min for 15 min. Supernatants were transferred into fresh tubes for analysis. The total quantity of protein in the lungs was measured using a BCA protein assay kit provided by KenGen Biotech Company, Nanjing, China, with protein concentration expressed as mg/mL. The concentrations of HGF, tumor necrosis factor- α (TNF-α), interleukin 8 (IL-8), interleukin 1 beta (IL-1β), interleukin 10 (IL-10) and myeloperoxidase (MPO) were measured using the respective ELISA kits (R&D systems Inc, USA). The absorbance at 450 nm was measured using a Biokinetics microplate reader ModelEL340 (Biotek Instruments, USA). The lung tissue levels of HGF, TNF-α, IL-8, IL-1β and IL-10 were expressed as ng/g protein.

### Real-time PCR for TLRs

Total RNA was extracted from lung tissues using a Qiagen RNeasy mini kit according to the manufacturer's instructions. RNA concentration was quantified and analyzed for purity (A260:280 ratio) using standard spectrophotometry (Biophotometer; Eppendorf, Hamburg, Germany). Real-time PCR was conducted using SYBR Green Master Mix kit (Bio-Rad) in a Bio-Rad C1000 thermal cycler. The following primers were used: TLR2 (forward: 5'-TCT GCT GTG CCC TTC TCC TGT TGA-3'; reverse: 5'-GGC CGC GTC GTT GTT CTC GT-3'); TLR4 (forward: 5'-AGC CGG AAG GTT ATT GTG GTA GT-3'; reverse: 5'-TGC CGT TTC TTG TTC TTC CTC T-3'); TLR7 (forward: 5'-TGC CAC CTA ATT TAC TAG AGC TCT ATC TTT AT-3'; reverse: 5'- TAG GTC AAG AAC TTG CAA CTC ATT G-3'); TLR9 (forward: 5'-GCA ATG GAA AGG ACT GTC CAC TTT GTG-3'; reverse: 5'-ATC GCC TTC TAT CGC CTT CTT GAC GAG-3'). Relative gene expression was determined using the 2^-ΔΔCt^ method with mRNA expression normalized to GAPDH mRNA levels.

### Western blot analysis

Protein concentrations in lung tissue extracts were quantified using a Bradford assay. Approximately 40 µg of protein were separated on 10% SDS-PAGE gels then transferred to PVDF membranes. After blocking with 10% non-fat milk, the membranes were incubated with rabbit anti-TLR4, anti-NF-κB and anti-cleaved-caspase-3 antibodies (1:1000 dilution; Santa Cruz) at 4ºC overnight. After three 10-min washes, the membranes were incubated with goat anti-rabbit IgG (1:5000 dilution) for 1 h at room temperature. Positive signals were developed using an ECL kit (Amersham Pharmacia Biotechnology Inc., Milpitas, CA), using anti-β-actin antibody (1:2000 dilution; Santa Cruz, CA, USA) as an internal control.

### Statistical analysis

Data analysis was performed using GraphPad Prism 5 software. The data were analyzed using normality and homogeneity tests of variance. Normally-distributed data were expressed as means ± SD. Values in multiple groups were compared using a one-way analysis of variance (ANOVA) and a Student-Newman-Keuls (SNK) test was used for pairwise comparison. A value of P <0.05 was considered statistically significant.

## Results

### Characterization of MSC-derived exosomes

**Figure 1:**
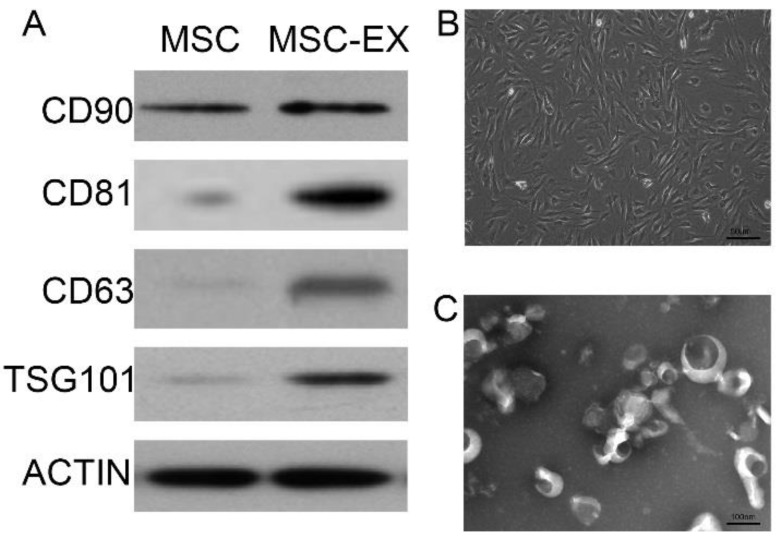
** Characterization of MSC-derived exosomes.** (A) Immunophenotype of bone marrow MSCs and MSC-derived exosomes (MSC-EX). Cells and exosomes were labeled with antibodies specific for the rat surface antigens indicated, then assessed by Western blot analysis. β-actin was used as an internal control. (B) Electron micrograph of rat MSCs. (C) Transmission electron micrograph of exosomes released from MSCs.

MSC-derived exosomes were identified by Western blot analysis and transmission electron microscopy (Figures 1A and 1C). MSC-derived exosomes displayed positive expression of exosome markers, such as CD81, CD63 and TSG101 (Figure 1A). The typical spindle-shaped appearance of MSCs was observed using optical microscopy (Figure 1B). Transmission electron micrographs confirmed the presence of exosomes approximately 100 nm in diameter, as homogeneous spheroids (Figure 1C).

### MSC-derived exosomes alleviated injury in the lungs of rats with IIR

**Figure 2:**
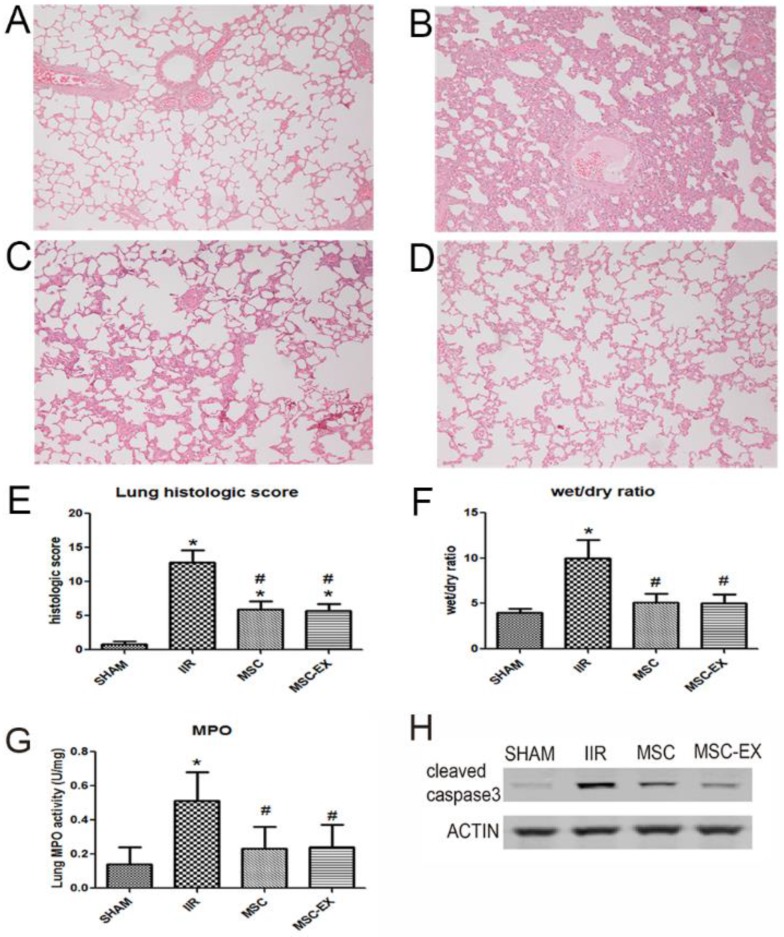
** MSC-derived exosomes alleviated lung injury induced by intestinal ischemia reperfusion.** Representative histologic appearance of lung tissue from: (A) SHAM group; (B) IIR group; (C) MSC group and (D) MSC-EX group 20 h after reperfusion following 75 min of intestinal ischemia (HE stained) (magnification×100). Bar graphs in (E), (F) and (G) display lung histological scores, wet/dry ratio and MPO activity, respectively. The data represent means ± standard deviation. n = 8 independent experiments. P values < 0.05 were considered statistically significant. * indicates p < 0.05 compared with SHAM group, # indicates p < 0.05 compared with IIR group. (H) Cleaved caspase-3 protein levels in lung assessed by Western blot analysis.

Histopathologic analysis demonstrated no significant pathologic findings in the SHAM group 20 h after reperfusion. In contrast, apparent edema and hemorrhage of the alveoli and mesenchyme, and inflammatory cell infiltration were observed in the IIR group that resulted in a significantly increased wet/dry ratio and lung histologic score (*p* < 0.01 IIR vs. SHAM, Figure 2). Only slight edema and hemorrhage of the alveoli and mesenchyme, and slight inflammatory cell infiltration were observed in the MSC and MSC-EX groups, with wet/dry ratios and histologic scores clearly decreased compared to the IIR injury group (both *p* < 0.05). MPO activity in the lung tissue, denoting neutrophil infiltration, was higher in the IIR group compared with the SHAM group, but lower in the MSC and MSC-EX groups. Apoptotic activity represented by cleaved caspase-3 increased as a result of IIR and decreased when treated with MSCs or MSC-derived exosomes (Figure 2).

### MSC-derived exosomes altered the balance of pro- and anti-inflammatory cytokines in lung tissue

**Figure 3:**
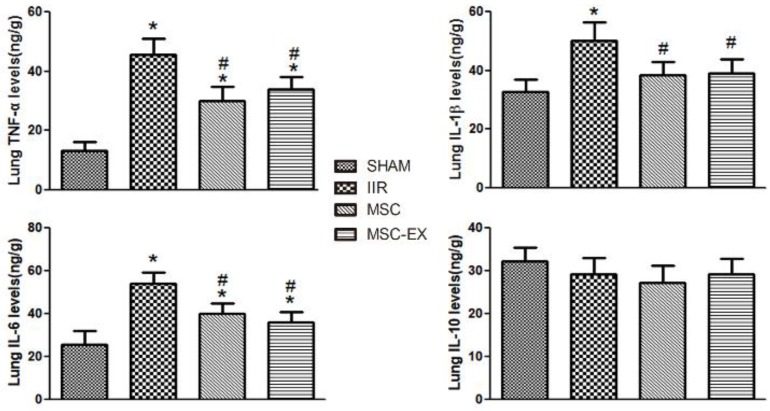
** MSC-derived exosomes modified anti-inflammatory and pro-inflammatory factor levels in lung tissue.** The lung tissue inflammatory markers TNF-α, IL-6, IL-1β and IL-10 levels were quantified 20 h after reperfusion. Data represent means ± standard deviation. n = 8 independent experiments. P values < 0.05 were considered statistically significant. * indicates p < 0.05 compared with SHAM group, # indicates p < 0.05 compared with IIR group.

Following IIR injury, pulmonary levels of pro-inflammatory cytokines including TNF-α, IL-6 and IL-1β increased considerably in the IIR group compared with the SHAM group but decreased in the MSC and MSC-EX groups in comparison. However, IL-10 levels decreased slightly in the IIR, MSC and MSC-EX groups compared with the SHAM group (Figure 3).

### MSC-derived exosomes decreased TLR4 and NF-κB levels in rat lung tissue

**Figure 4:**
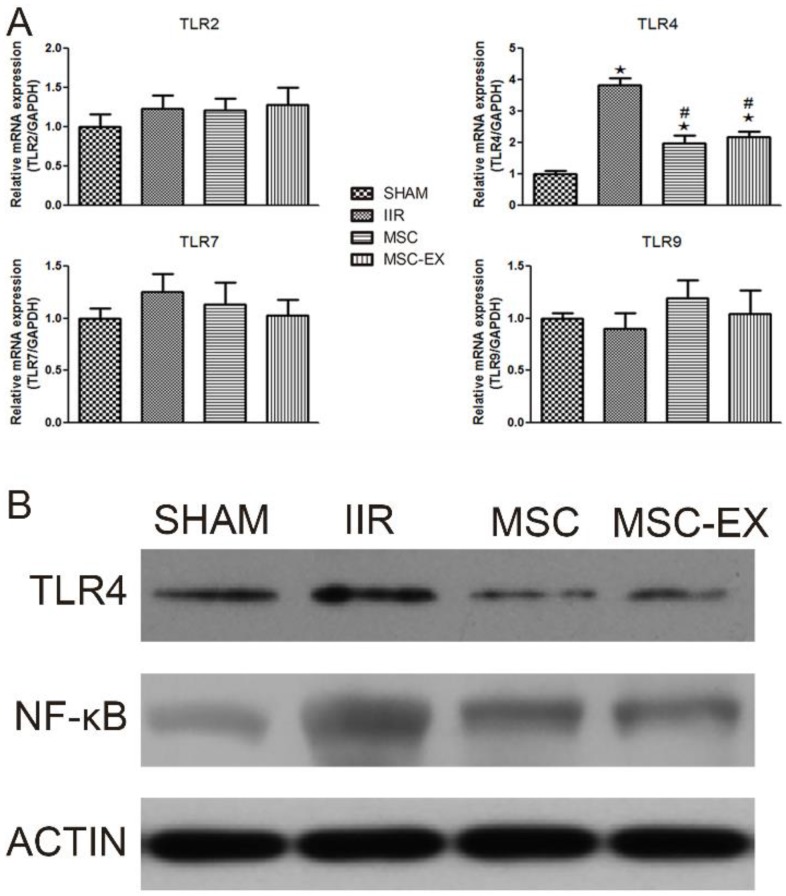
** MSC-derived exosomes decreased TLR4 and NF-κB levels in lung tissue.** Rats were sacrificed 20 h after reperfusion following 75 min of intestinal ischemia. (A) mRNA levels of TLR2, TLR4, TLR7 and TLR9 in lung tissue assessed by real time PCR. (B) TLR4 and NF- κB protein levels in lung tissue assessed by Western blot analysis. P values < 0.05 were considered statistically significant. * indicates p < 0.05 compared with SHAM group, # indicates p < 0.05 compared with IIR group.

The mRNA levels of relevant genes in lung tissue were quantified using RT-PCR. Among the TLR family, only TLR4 mRNA levels were up-regulated by IIR but down-regulated by MSC or MSC-EX treatment. Similarly, Western blot analysis also demonstrated that IIR increased the expression of TLR4 protein, in addition to NF- κB, while MSC and MSC-EX treatment decreased both TLR4 and NF-κB protein levels (Figure 4).

## Discussion

In the present study, we demonstrated that MSC-derived exosomes down-regulated TLR4 and NF-κB, and attenuated IIR-induced lung damage, apoptosis and inflammation. These results indicate that the protection afforded by MSC-derived exosomes against IIR-induced cell death and inflammation in lungs may be dependent on the modulation of the TLR4/NF-κB signaling pathway.

The murine model used in this study is both simple and reproducible and one which is the most relevant for acute occlusive mesenteric ischemia in clinical situations. Patients with intestine ischemia reperfusion injury often receive revascularization before being diagnosed accurately 1, 2. Furthermore, liquid resuscitation allowed experimental animals to survive until a future collection point (20 hours) 17. Similar to the reports of Cen *et al.* and McGinn *et al.*, a firm and significant difference in lung damage between groups was observed 20 h after IIR in this study 18, 19. Acute lung injury after IIR was clearly characterized as acute inflammation with apparent edema and hemorrhage of alveoli and mesenchyme with inflammatory cell infiltration in this study.

A limited number of treatment options, such as respiratory support, small dose corticosteroid and conservative fluid therapy, are currently considered for the treatment of acute lung injury (ALI) 6. However, the therapeutic effects remain unsatisfactory. Mesenchymal stem cells (MSCs) show considerable promise as a biological therapy for a diverse range of unmet medical needs, including acute lung injury 7, 9. MSCs have been demonstrated to accelerate recovery from lung injury induced by endotoxin, bleomycin and radiation 9. In this study, we confirmed that systemic MSC administration mitigated lung damage after IIR. In many studies the paracrine activity of MSCs has been implicated as the prime mechanism of their mode of action 12, 20. Exosomes are simple to isolate and safe to use and have been reported to be important in the mediation of paracrine actions 14, 15. Researchers have discovered that the secretion-based effects of MSCs are principally mediated by exosomes 15, 21. MSC-derived exosomes have been shown to mimic the protection against liver injury, myocardial infarction and lung injury in mouse models provided by intravenously-administered MSCs 11, 22, 23. However, no related reports have been published that indicate whether MSCs can further enhance the recovery of IIR-induced ALI or if treatment can be achieved with exosomes. For the first time, the present study has demonstrated that MSC-derived exosomes are capable of stabilizing alveoli and epithelial cells injured by IIR, thereby reducing inflammation and promoting the recovery of pulmonary function, similar to treatment with MSCs. Previous evidence has also shown that intravenous injection (IV) of MSCs is as effective at treating lung disease as intratracheal injection 24. This study also confirmed the efficacy of IV administration of MSCs and MSC-derived exosomes for treating injured lungs. These suggest that exosomes may home to the site of inflammation. In contrast to cell therapy, exosomes have been shown to exhibit decreased immunogenicity and oncogenic potential and have excellent biocompatibility for the delivery of their bioactive contents 23, 25. Thus, the use of MSC-derived exosomes may become a promising non-cellular alternative to MSC transplantation. Three clinical trials of therapeutic interventions utilizing MSC-derived exosomes are currently ongoing (NCT03384433, NCT02138331 and NCT03437759).

Exosomes have been reported to mediate intercellular communication by transferring proteins, lipids and genomic material including mRNAs, miRNAs and snRNAs between source and target cells 14, 21. Recent studies have shown that biomolecules in exosomes can be recognized by Toll like receptors (TLRs) leading to differential inflammatory and immunomodulatory effects on target cells 26-28. In this study, we found that TLR4 and not TLR2, TLR7 or TLR9, was up-regulated by IIR injury and ameliorated by MSC and MSC-derived exosome treatment, associated with reduced lung injury. This was consistent with previous studies demonstrating that disruption of TLR4 attenuates lung inflammation and injury 29, 30. Although therapies directly targeting TLR4 showed promise in preclinical studies of shock and respiratory failure, they failed to demonstrate efficacy in clinical trials 31. Results of this study expose a further possible therapeutic opportunity to target TLR4 through administration of MSC-derived exosomes. In addition, the expression pattern of NF-κB protein was similar to that of TLR4 in this study, suggesting the involvement of the TLR4/NF-κB pathway in the protection by MSC-derived exosomes of IIR-induced lung injury. TLR4/NF-κB signaling had been reported to be a key pathway regulating the production of proinflammatory mediators in a mouse model of intestinal I/R induced lung damage 32. TNF-α, IL-1β and IL-6 are downstream targets of the NF-κB signaling pathway 33, 34. Reduced expression of pulmonary TNF-α, IL-1β and IL-6 in MSC-treated and exosome-treated groups in this study confirmed the hypothesis that inactivation of TLR4-NF-κB signaling is the underlying mechanism of protection that occurs in MSC-derived exosomes in IIR-induced lung injury. Although IL-10 has been reported to be a proinflammatory mediator in a murine model of IIR-induced lung damage, its expression was not affected by the administration of MSCs or MSC-derived exosomes 35. Moreover, inactivation of TLR4/NF-κB signaling was accompanied by down-regulation of a key protease in the apoptotic cascade, caspase-3, suggesting that protection by MSC-derived exosomes against ALI may involve inhibition of apoptosis in pulmonary microvascular epithelial cells through TLR4/NF-κB signaling 36.

There were some limitations to this study. Firstly, no constituents of exosomes derived from MSCs were identified. Secondly, the specific biomolecules inside the exosomes which interact with TLR4 leading to subsequent regulation of inflammation, have not been clarified. Further studies are planned to answer these questions.

In summary, the current study demonstrated that exosomes released by MSCs exhibit a protective capacity similar to that of MSCs in lung injury triggered by intestinal ischemia reperfusion in a rat model. Downregulation of TLR4/NF-κB signaling underlays the protection of MSC-derived exosomes against IIR-induced cell death and inflammation in lungs. These findings add new insights to the potential mechanism of exosome-based treatment, and provide a potential strategy against IIR-mediated acute lung injury by use of exosomes as a non-cellular alternative to MSC transplantation.

## References

[1] Oldenburg WA, Lau LL, Rodenberg TJ (2004). Acute mesenteric ischemia: a clinical review. Arch Intern Med.

[2] Clair DG, Beach JM (2016). Mesenteric Ischemia. N Engl J Med.

[3] Rubenfeld GD, Caldwell E, Peabody E (2005). Incidence and outcomes of acute lung injury. N Engl J Med.

[4] Mallick IH, Yang W, Winslet MC (2004). Ischemia-reperfusion injury of the intestine and protective strategies against injury. Dig Dis Sci.

[5] Hassoun HT, Kone BC, Mercer DW (2001). Post-injury multiple organ failure: the role of the gut. Shock.

[6] Fan E, Brodie D, Slutsky AS (2018). Acute Respiratory Distress Syndrome: Advances in Diagnosis and Treatment. JAMA.

[7] Brooke G, Cook M, Blair C (2007). Therapeutic applications of mesenchymal stromal cells. Semin Cell Dev Biol.

[8] Ding DC, Shyu WC, Lin SZ (2011). Mesenchymal stem cells. Cell Transplant.

[9] Horie S, Laffey JG (2016). Recent insights: mesenchymal stromal/stem cell therapy for acute respiratory distress syndrome.

[10] Liu J, Pan G, Liang T (2014). HGF/c-Met signaling mediated mesenchymal stem cell-induced liver recovery in intestinal ischemia reperfusion model. Int J Med Sci.

[11] Tan CY, Lai RC, Wong W (2014). Mesenchymal stem cell-derived exosomes promote hepatic regeneration in drug-induced liver injury models. Stem Cell Res Ther.

[12] Ionescu L, Byrne RN, van Haaften T (2012). Stem cell conditioned medium improves acute lung injury in mice: in vivo evidence for stem cell paracrine action. Am J Physiol Lung Cell Mol Physiol.

[13] Rager TM, Olson JK, Zhou Y (2016). Exosomes secreted from bone marrow-derived mesenchymal stem cells protect the intestines from experimental necrotizing enterocolitis. J Pediatr Surg.

[14] Raposo G, Stoorvogel W (2013). Extracellular vesicles: exosomes, microvesicles, and friends. J Cell Biol.

[15] Phinney DG, Pittenger MF (2017). Concise Review: MSC-Derived Exosomes for Cell-Free Therapy. Stem Cells.

[16] Derks CM, Jacobovitz-Derks D (1977). Embolic pneumopathy induced by oleic acid. A systematic morphologic study. Am J Pathol.

[17] Huang P, Liu D, Gan X (2012). Mast cells activation contribute to small intestinal ischemia reperfusion induced acute lung injury in rats. Injury.

[18] Cen C, Yang WL, Yen HT (2016). Deficiency of cold-inducible ribonucleic acid-binding protein reduces renal injury after ischemia-reperfusion. Surgery.

[19] McGinn JT, Aziz M, Zhang F (2018). Cold-inducible RNA-binding protein-derived peptide C23 attenuates inflammation and tissue injury in a murine model of intestinal ischemia-reperfusion. Surgery.

[20] Biancone L, Bruno S, Deregibus MC (2012). Therapeutic potential of mesenchymal stem cell-derived microvesicles. Nephrol Dial Transplant.

[21] Chen W, Huang Y, Han J (2016). Immunomodulatory effects of mesenchymal stromal cells-derived exosome. Immunol Res.

[22] Lai RC, Arslan F, Lee MM (2010). Exosome secreted by MSC reduces myocardial ischemia/reperfusion injury. Stem Cell Res.

[23] Monsel A, Zhu YG, Gudapati V (2016). Mesenchymal stem cell derived secretome and extracellular vesicles for acute lung injury and other inflammatory lung diseases. Expert Opin Biol Ther.

[24] Rojas M, Xu J, Woods CR (2005). Bone marrow-derived mesenchymal stem cells in repair of the injured lung. Am J Respir Cell Mol Biol.

[25] Jing H, He X, Zheng J (2018). Exosomes and regenerative medicine: state of the art and perspectives. Transl Res.

[26] Liu J, Jiang M, Deng S (2018). miR-93-5p-Containing Exosomes Treatment Attenuates Acute Myocardial Infarction-Induced Myocardial Damage. Mol Ther Nucleic Acids.

[27] Kojima M, Gimenes-Junior JA, Chan TW (2018). Exosomes in postshock mesenteric lymph are key mediators of acute lung injury triggering the macrophage activation via Toll-like receptor 4. FASEB J.

[28] Seo W, Eun HS, Kim SY (2016). Exosome-mediated activation of toll-like receptor 3 in stellate cells stimulates interleukin-17 production by gammadelta T cells in liver fibrosis. Hepatology.

[29] Victoni T, Coelho FR, Soares AL (2010). Local and remote tissue injury upon intestinal ischemia and reperfusion depends on the TLR/MyD88 signaling pathway. Med Microbiol Immunol.

[30] Zhu Q, He G, Wang J (2017). Down-regulation of toll-like receptor 4 alleviates intestinal ischemia reperfusion injury and acute lung injury in mice. Oncotarget.

[31] Rice TW, Wheeler AP, Bernard GR (2010). A randomized, double-blind, placebo-controlled trial of TAK-242 for the treatment of severe sepsis. Crit Care Med.

[32] Ben DF, Yu XY, Ji GY (2012). TLR4 mediates lung injury and inflammation in intestinal ischemia-reperfusion. J Surg Res.

[33] Liu T, Zhang L, Joo D (2017). NF-kappaB signaling in inflammation.

[34] Zhang Q, Lenardo MJ, Baltimore D (2017). 30 Years of NF-kappaB: A Blossoming of Relevance to Human Pathobiology. Cell.

[35] Yang Z, Zhang XR, Zhao Q (2018). Knockdown of TNFalpha alleviates acute lung injury in rats with intestinal ischemia and reperfusion injury by upregulating IL10 expression. Int J Mol Med.

[36] Li Y, Cao Y, Zeng Z (2015). Angiotensin-converting enzyme 2/angiotensin-(1-7)/Mas axis prevents lipopolysaccharide-induced apoptosis of pulmonary microvascular endothelial cells by inhibiting JNK/NF-kappaB pathways. Sci Rep.

