# Exosomes Released by Bone Marrow Mesenchymal Stem Cells Attenuate Lung Injury Induced by Intestinal Ischemia Reperfusion via the TLR4/NF-κB Pathway: Erratum

**DOI:** 10.7150/ijms.86924

**Published:** 2023-07-06

**Authors:** Jianpei Liu, Tufeng Chen, Purun Lei, Xiao Tang, Pinjie Huang

**Affiliations:** 1 Department of Gastrointestinal Surgery, The Third Affiliated Hospital of Sun Yat-sen University, Guangzhou, China, 510630.; 2 Department of Anesthesiology, The Third Affiliated Hospital of Sun Yat-sen University, Guangzhou, China, 510630.

When reviewing the previous work, we realized the images of the original Figure 1 of our article were incorrectly assembled. The image of the Western blot for CD90 in the original Figure 1A was misused due to a mislabeling error. We sincerely apologize for this error. Figure 1 should be corrected as follows. The authors confirm that the correction made in this erratum does not affect the original conclusions.

## Figures and Tables

**Figure 1:**
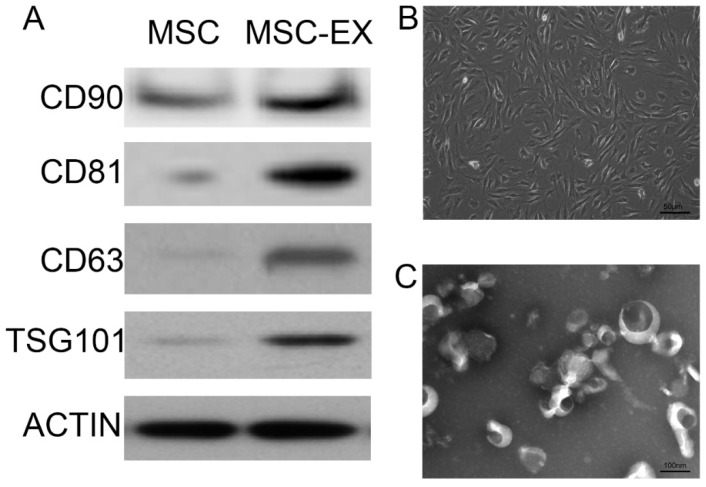
Characterization of MSC-derived exosomes. (A) Immunophenotype of bone marrow MSCs and MSC-derived exosomes (MSC-EX). Cells and exosomes were labeled with antibodies specific for the rat surface antigens indicated, then assessed by Western blot analysis. β-actin was used as an internal control. (B) Electron micrograph of rat MSCs. (C) Transmission electron micrograph of exosomes released from MSCs.

